# ZrO_2_ Based Multilayered Stacks with Al_2_O_3_, Y_2_O_3_ or La_2_O_3_ Interlayers for SiC Power Devices

**DOI:** 10.1021/acsami.5c00947

**Published:** 2025-05-02

**Authors:** Sandra Krause, Aleksey Mikhaylov, Uwe Schroeder, Thomas Mikolajick, Andre Wachowiak

**Affiliations:** † TU Dresden, 01062 Dresden, Germany; ‡ Infineon Technologies Austria, Siemensstraße 2, 9500 Villach, Austria; § NaMLab gGmbH, Nöthnitzer Straße 64, 01187 Dresden, Germany

**Keywords:** dielectrics, nanolaminates, silicon carbide, high-k, multilayer

## Abstract

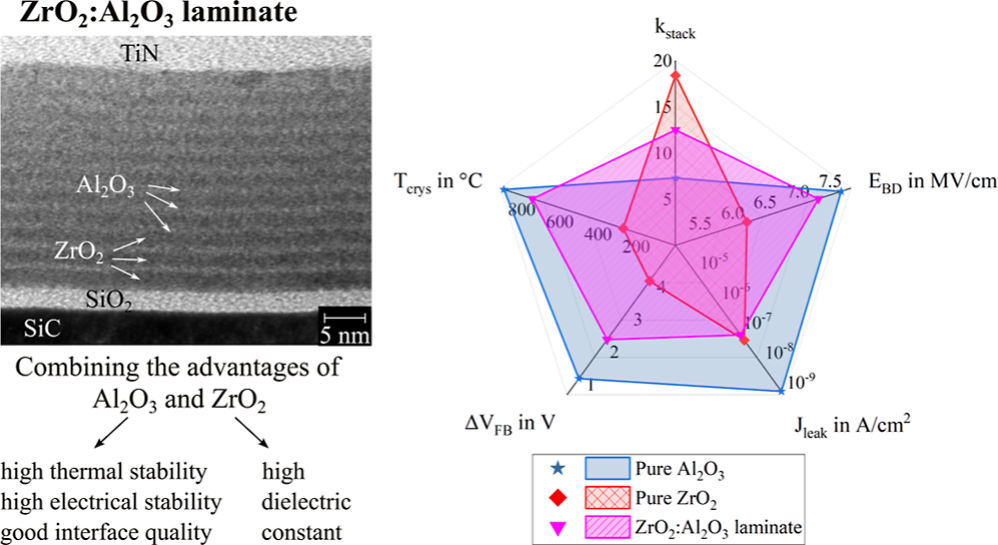

ZrO_2_ is a promising high-*k* dielectric for SiC power devices due to its favorable bandgap alignment with SiC. However, it exhibits low breakdown fields and excessively high leakage currents for thicker layers. This paper presents an approach to suppress this leakage current by integrating thin interlayers of Al_2_O_3_, Y_2_O_3_, or La_2_O_3_ into the ZrO_2_ film. These interlayers significantly reduce the charge carrier transport through ZrO_2_ films and, thereby, the leakage current of the stack. Among the investigated interlayers, Al_2_O_3_ shows the most pronounced effect, reducing the leakage of ZrO_2_-based thick films by 2 orders of magnitude and achieving a breakdown field of 7.4 MV/cm. This is comparable to the value measured for pure Al_2_O_3_ (7.7 MV/cm). These improvements can be attributed to the amorphous nature of the laminated oxide as the crystallization temperature could be increased from 350 °C for pure ZrO_2_ up to 750 °C for the nanolaminate. Notably, the dielectric constant of this optimized stack is 13, which is twice as high as that of pure Al_2_O_3_. No additional charge trapping due to the interlayers was detected by Capacitance–Voltage hysteresis measurements. Furthermore, by additional optimization of the stack’s deposition conditions, the charge trapping was reduced by 50% compared to pure ZrO_2_ films.

## 1 Introduction

In recent years, silicon carbide (SiC) has emerged as a superior semiconductor material in high-power applications, surpassing silicon (Si). The higher electric breakdown field of SiC results in higher blocking voltage and lower on-state losses in SiC devices.^1,2^ However, current SiC technology is constrained by silicon oxide (SiO_2_) properties. Specifically, the higher electric fields present in SiC devices compared to Si induces excessive electric fields within the SiO_2_ leading to its breakdown. This issue highlights the urgent need for novel dielectric materials for future SiC devices.^3−5^ Dielectric materials with high dielectric constant (*k*) have been extensively researched as a replacement for ultrathin SiO_2_ in Si technologies.^2,5,6^ In SiC power devices, these high-*k* dielectrics are a promising element for enabling the full potential of the SiC properties. A higher k value of the dielectric leads to a stronger internal polarization, which reduces the internal electric field. Furthermore, the ratio between the dielectric constants of dielectric and the semiconductor determines the distribution of the electric fields within the device as the vertical component of the electric displacement field is consistent at any interface. For an oxide with high dielectric constant, the major part of the electric field is pushed into the SiC during depletion. As a result, the effective electric field within the oxide is lower.^3,7,8^ In the context of SiC technologies, the desirable range of the dielectric constant typically spans from 8 (pure Al_2_O_3_) to 40 (crystalline ZrO_2_ or HfO_2_). Beyond this range, the complexities in device integration can outweigh the potential benefits of incorporating high-*k* materials.^3,4,9^

There are various options for high-*k* materials. For integration into semiconductor devices there is a particular focus on binary metal oxides.^3,4,9^ Hafnium dioxide (HfO_2_) thin films have already been successfully applied in logic devices. However, the challenge remains in developing thick films greater than 10 nm with sufficient electrical properties for power devices.^2^ Aluminum oxide (Al_2_O_3_) has been extensively studied due to its wide bandgap of 7 eV, which is essential for providing a low leakage current and a high electric breakdown field. Additionally, its high crystallization temperature (*T*_crys_) of 900 °C indicates good processing compatibility with high temperature back-end-of-line processes.^3^ In contrast, its relatively low *k* value of 8 suggests that other materials might offer greater benefits. Materials of interest include aluminum nitride (AlN), yttrium oxide (Y_2_O_3_), zirconium dioxide (ZrO_2_), lanthanum oxide (La_2_O_3_) or alloys such as lanthanum aluminate (LaAlO). ZrO_2_ is a particularly promising candidate due to its chemical similarity to HfO_2_.^3^ However, the conduction band offset and the *k* value of crystalline ZrO_2_ are higher than for HfO_2_. As reported by Siddiqui et al., ZrO_2_ has a bandgap of 5.6 eV (5.7 eV for HfO_2_) that aligns well with the bandgap of SiC, offering a comparatively high conduction band offset of 1.82 eV.^3^ This conduction band offset is crucial for preventing electron tunneling through the oxide in the on-state of the power device. The *k* value for ZrO_2_ reported in the literature ranges from 20 for monoclinic crystalline ZrO_2_ to 40 for tetragonal crystalline films.^10^

**Figure 1:**
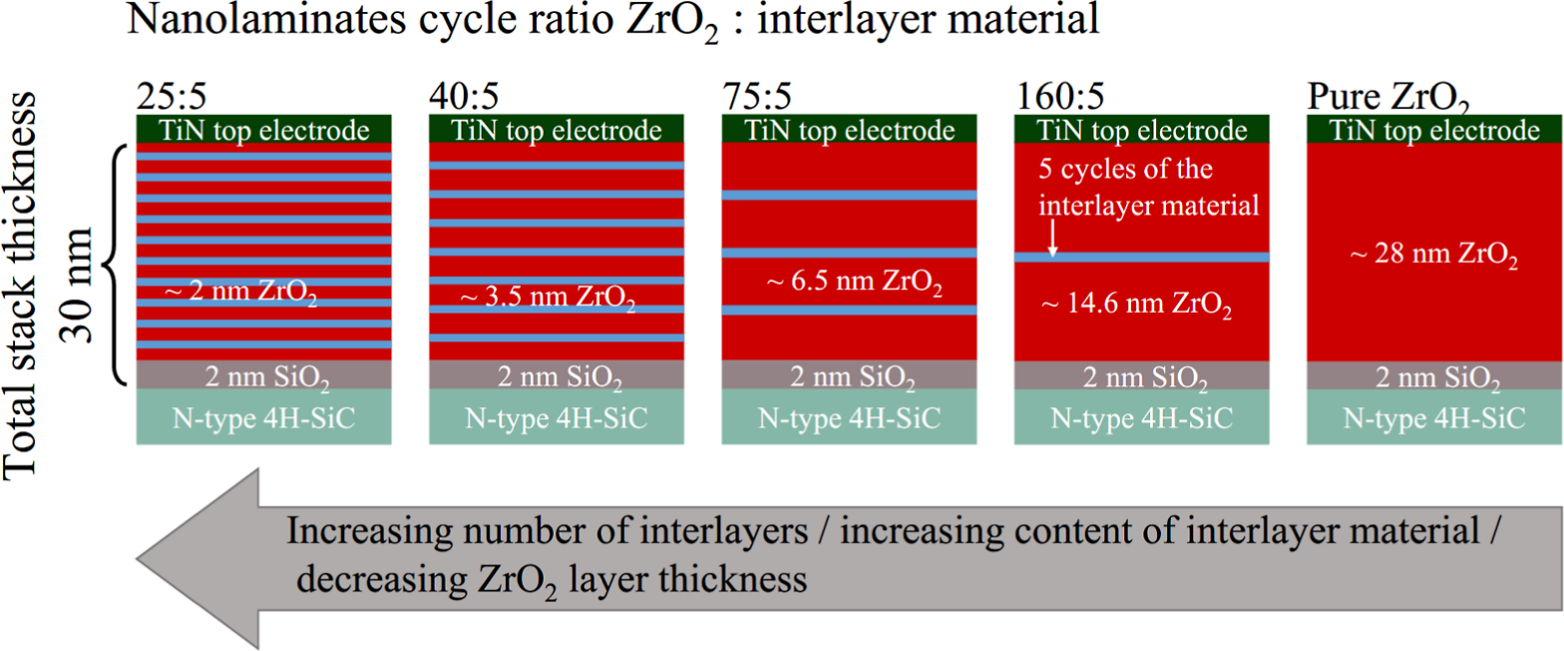
Schematic representation of the prepared samples.

The challenge for ZrO_2_ arises when increasing the oxide thickness: thicker films exhibit a significantly increased leakage current at the same electric field, which is unacceptable for fabricating reliable devices.^11^ This effect is well-known for many dielectric materials including SiO_2_ and can be ascribed effects like impact ionization that can be neglected for thin films but becomes increasingly relevant for thick oxides. This paper presents an approach to suppress this leakage current in ZrO_2_ by inserting monolayers of another dielectric material into the oxide stack. The concept of laminating oxides was utilized by several researchers leading to improved electrical parameters.^12−14^ The nanolaminates leverage the low leakage and high crystallization temperature of thin films by constructing a stack composed of multiple individual thin ZrO_2_ layers, each separated by the monolayers (Figure 1). These interlayers reduce the carrier transport through the oxide film by interrupting leakage paths. In addition, interlayers of a material with larger bandgap increase the band offset between SiC and ZrO_2_ which prevents the electron transport through the oxide and increases the electric breakdown stability. Furthermore, the thermal budget applied to the oxide during device processing and operation is one of the major challenges for integrating high-*k* materials. Most high-*k* materials crystallize at temperatures below 500 °C. However, crystalline oxides typically exhibit higher leakage currents and lower breakdown fields than their amorphous counterparts. Therefore, increasing the crystallization temperature, particularly for stacks thicker than 15 nm, is an additional goal that we targeted with ZrO_2_-based nanolaminates.

Three different interlayer materials were investigated: Al_2_O_3_, Y_2_O_3_, and La_2_O_3_. The properties of these materials are summarized in Table 1. It is important to note that the properties can vary greatly depending on the deposition parameters and postdeposition treatment. Despite these variations, Table 1 provides an initial overview of the benefits and drawbacks of each material.

**Table 1:** Properties of the Investigated Dielectric Materials^a^

	ZrO_2_	Al_2_O_3_	Y_2_O_3_	La_2_O_3_
Bandgap	5.6 eV^3^	7.0 eV^3^	5.5 eV^21^	5.45 eV^3,22^
	5.8 eV^20^			5.8 eV^23^
Conduction band offset to SiC	1.82 eV^3^	1.9 eV^3^	1.96 eV^24^	0.99 eV^3^
Dielectric constant *k* amorphous	16–26^20^	6^25^	14–18	21^27^
			15^26^	27^28^
Dielectric constant *k* crystalline	32 (t/c)^10^	9.5^25^	15 (c)^29^	27 (h)^23^
	25 (m)^29^			30 (h/c)^29^
Crystallization temperature *T*_crys_ for films >15 nm	300–400 °C^30^	900 °C^3,31^	<300 °C^32^	500–600 °C^22^
		1150 °C^25^	400–600 °C^33^	
Valency	+4^34^	+3^34^	+3^34^	+3^34^
Atomic radius	155 pm^35^	125 pm^35^	180 pm^35^	195 pm^35^

a The shortcuts for the crystallographic phases are t: tetragonal, c: cubic, m: monoclinic and h: hexagonal.

The large bandgap of Al_2_O_3_ makes it a promising candidate for reducing the charge carrier transport through the ZrO_2_. Several studies^15,16^ have shown that a single Al_2_O_3_ interlayer can significantly reduce the leakage in ZrO_2_ thin films to a stack thickness of 6 nm. This principle was expanded to create ZrO_2_:Al_2_O_3_ laminates with increased *T*_crys_.^14^ However, detailed leakage and breakdown measurements have not been presented. Lo Nigro et al. demonstrated that Al_2_O_3_:HfO_2_ nanolaminates have increased *T*_crys_ and reduced leakage current for stacks with thicknesses up to 30 nm. However, the relatively low k value of Al_2_O_3_ increases the equivalent oxide thickness (EOT) of the stack.^13^ Y_2_O_3_ and La_2_O_3_ have higher k values, which could enable the creation of nanolaminates without increasing EOT. Seo et al. found that, unlike Al atoms, Y atoms diffuse into the ZrO_2_ and do not form a closed interlayer. As a result, the Y_2_O_3_ interlayers cannot inhibit the ZrO_2_ crystallization but still reduce the leakage current. The k value of the stack remained high.^16^ Ginestra et al. showed that thicker Y_2_O_3_ interlayers (1.8 nm) can separate the ZrO_2_ film and shift the onset of the crystallization process toward higher temperatures.^17^ A similar effect could be expected from La_2_O_3_, as researchers reported high k value and high *T*_crys_.^18^ Lee et al., e.g., presented 6 nm thick La_2_O_3_:HfO_2_ stacks with thermal stability up to 950 °C and enhanced electrical properties.^19^ While these publications provide valuable insights, there is still a lack of comprehensive studies that expand the principle of interlayers creating nanolaminates with thickness above 20 nm, particularly for ZrO_2_-based stacks. Furthermore, many of the previous results have been obtained on Si substrates and can only partially be transferred to the wide bandgap material SiC, as the functional requirements on the dielectric differ a lot for SiC electronics compared to Si. Our work aims to go beyond the mentioned studies and combine their results to achieve better electrical stability in ZrO_2_ films up to 45 nm thickness.

## 2 Experimental Section

Dielectric stacks with a target thickness of 30 nm were fabricated by atomic layer deposition (ALD) using an Oxford Instruments OpAL reactor. Detailed deposition conditions can be found in Table 2 and in Alcala et al.^36^ The target thickness of 30 nm was chosen as it is sufficient to achieve the bulk limit in electrical parameters and crystallization temperature, yet can still be deposited using the high-quality but time-consuming ALD processes within a reasonable time frame. The number of interlayers varied between 0 and 10. As the number of interlayers increased, the number of ZrO_2_ pulses was adjusted to reduce the thickness of the individual ZrO_2_ layers, ranging from 2 to 15 nm. This adjustment ensured that the total stack thickness remained constant at ∼28 nm. A schematic representation of the investigated stacks is shown in Figure 1. Each interlayer consisted of 5 ALD cycles to ensure the formation of thin, but complete (pinhole free) layers. This resulted in ALD cycle ratios for ZrO_2_:interlayer of 25:5, 40:5, 75:5, and 160:5. The deposition temperature was 250 °C. O_3_ was used as oxidant because O_3_ deposited films displayed the most promising results in previous experiments.^36^ The stacks were deposited on top of a 2–3 nm thick thermally grown SiO_2_ on a 4H-SiC substrate. As highlighted in various studies, the inclusion of this thin interfacial layer is beneficial for ALD growth, band alignment, channel mobility, and frequency dispersion.^37−40^

**Table 2:** Overview over the ALD Deposition Conditions

Material	Precursor
ZrO_2_	ZyALD from Air liquide
Al_2_O_3_	TMAl SSG from AkzoNobel
La_2_O_3_	LOLA PRIME from Air Liquide
Y_2_O_3_	ARYA from Air Liquide

A 10 nm thick titanium nitride (TiN) film was sputtered as top electrode. Following the TiN deposition, the samples were annealed at 500 °C for 1 min in argon ambient to simulate a thermal budget required for power device fabrication. The layer thicknesses were determined by X-ray reflectometry (XRR) or transmission electron microscopy (TEM). The crystallographic phase of the high-*k* film was determined from the peak positions in grazing-incidence X-ray diffraction measurements (GIXRD). For the poly crystalline films the out-of-plane grain diameter was estimated from the peak width in the GIXRD spectra using the Debye–Scherrer relation.^41^ Capacitance–voltage (C(V)) measurements were carried out to determine the dielectric constant (*k* value) and the trapping behavior. The *k* value was calculated from the capacitance of the metal-oxide-semiconductor (MOS) structure in accumulation. The leakage current density and the electric breakdown field (*E*_BD_) were determined using current–voltage (*I*(*V*)) measurements. *E*_BD_ was defined as the electric field at which the leakage current density exceeds a limit of 0.01 mA/cm^2^. For comparison purposes, a value for the leakage current density (*J*_leak_) was extracted at an applied field of 2 MV/cm. The error value for each parameter was obtained by statistical analysis of measurements on 3–10 devices and by the physical limits of the measurement equipment.

## 3 Results

### 3.1 Current–Voltage Measurements I(V)

**Figure 2:**
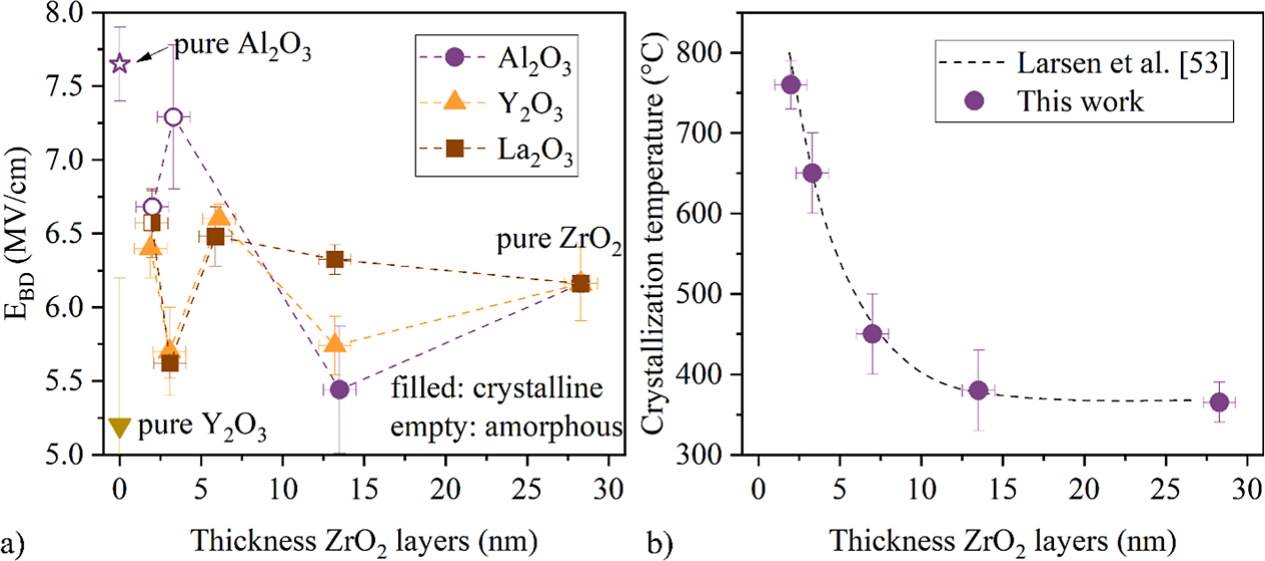
(a) Electric breakdown field *E*_BD_ of the samples as a function of the thickness of the individual ZrO_2_ layers within the laminates. Samples with pure Al_2_O_3_ and Y_2_O_3_ were added for reference. All laminates exhibit an increase in *E*_BD_ compared to pure ZrO_2_. (b) Crystallization temperature *T*_crys_ for the ZrO_2_:Al_2_O_3_ laminates as a function of the ZrO_2_ layer thickness revealing an exponential increase of *T*_crys_ if the ZrO_2_ thickness in the laminates is reduced.

Most nanolaminates exhibit an increased E_BD_ compared to pure ZrO_2_ films (Figure 2a). This effect is most pronounced for the samples with Al_2_O_3_ interlayers. Specifically, the sample with a cycle ratio of 40:5 shows an *E*_BD_ nearly as high as that of pure Al_2_O_3_. The *k* value of this material is 16, which is more than twice the *k* value of pure Al_2_O_3_ (*k* = 7). Alternatively stated, we have developed a stack with an electrical strength close to that of Al_2_O_3_ but more than twice the dielectric constant of Al_2_O_3_. The enhanced electrical stability can be attributed to the amorphous structure of the sample. Crystalline oxides provide leakage paths for charge carriers along crystal grain boundaries, leading to a high leakage and early breakdown. The samples with multiple Al_2_O_3_ interlayers are amorphous after the 500 °C anneal, resulting in higher *E*_BD_. To validate the connection between crystallinity and breakdown field, samples with pure ZrO_2_ films without annealing were fabricated. These ZrO_2_ films are amorphous and exhibited an *E*_BD_ that is 0.6 MV/cm higher than the breakdown field for their annealed (and crystalline) counterparts.

**Figure 3:**
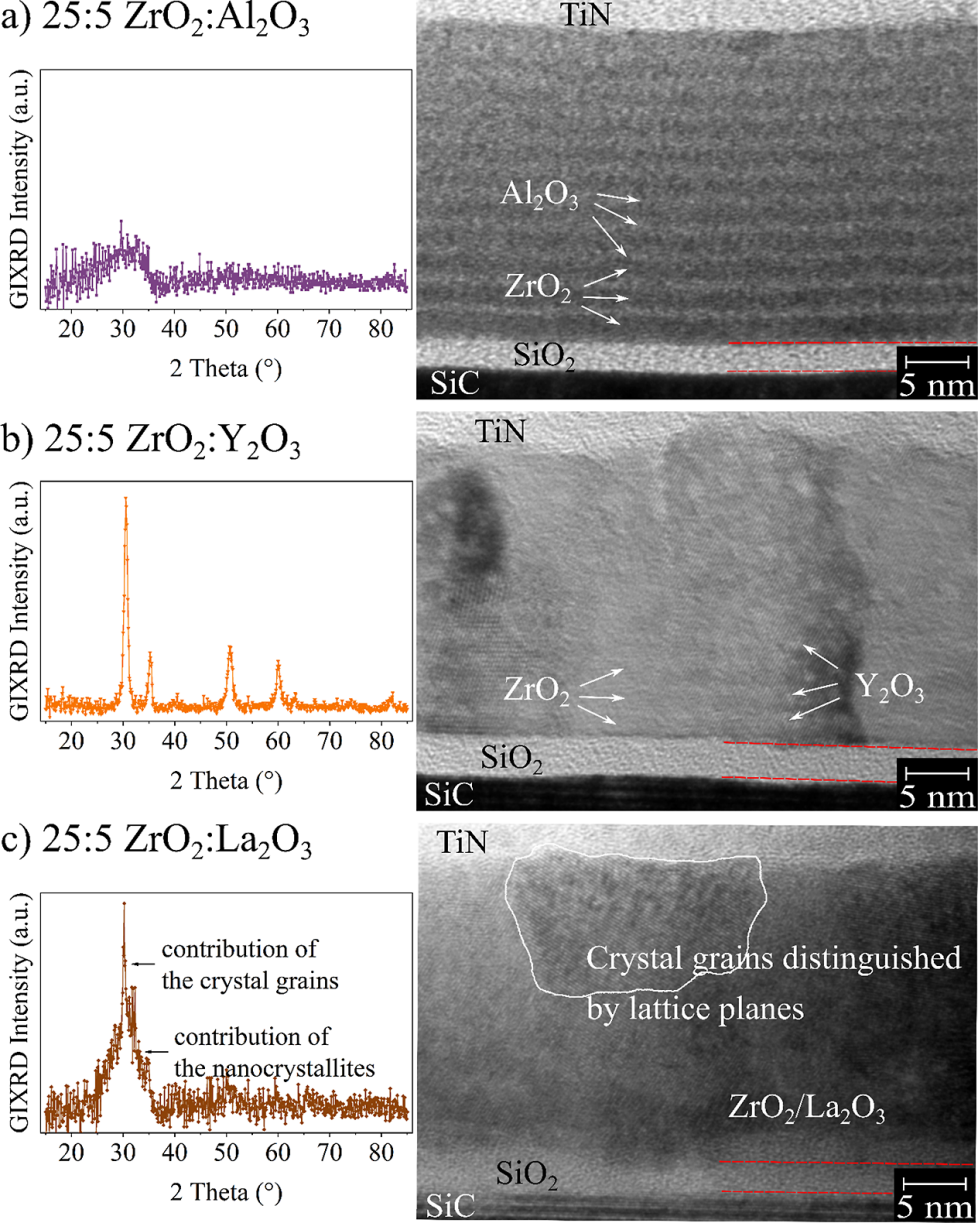
GIXRD scans (left) and TEM images (right) of the (a) ZrO_2_:Al_2_O_3_ (b) ZrO_2_:Y_2_O_3_ and (c) ZrO_2_:La_2_O_3_ nanolaminates with ALD cycle ratio 25:5, annealed at 500 °C for 1 min in Argon atmosphere.

The crystallization temperatures (*T*_crys_) for the ZrO_2_:Al_2_O_3_ nanolaminates were obtained by subsequent annealing experiments in combination with GIXRD measurements and are shown in Figure 3b. With increasing number of interlayers, i.e. with decreasing thickness of the ZrO_2_ films, *T*_crys_ increases exponentially. This can be attributed to the higher *T*_crys_ for thinner films.^30,42^ The measured temperatures coincide well with the values presented by Larsen et al.,^30^ who studied ZrO_2_ nanolayered with silica. We achieve the same results with Al_2_O_3_ interlayers, which suggests that Al_2_O_3_ completely interrupts the ZrO_2_ bulk. This is confirmed by the TEM image in Figure 3a.

The increasing *T*_crys_ in thin layers is a consequence of the energy relations during crystallization. The effective specific energy required to form an amorphous/crystalline interface is defined as the difference in the Gibbs free energy between the nuclei in the amorphous and crystalline phases. In thin layers, the surface to bulk ratio changes with layer thickness, thereby increasing the energy necessary for crystallization. This results in a higher *T*_crys_ for thin oxide layers. There is a critical thickness below which no crystallization occurs.^42^ For the sample with a cycle ratio of 25:5, corresponding to ZrO_2_ layers of approximately 2 nm thickness, *T*_crys_ increased from 375 °C (pure ZrO_2_) up to 750 °C.

The 25:5 Y_2_O_3_:ZrO_2_ laminate is crystalline after the anneal as can be seen from the GIXRD spectra and the TEM image in Figure 3b. The Y_2_O_3_:ZrO_2_ laminates do not exhibit an increased *T*_crys_, which can be attributed to the larger atomic radii of Y atoms and their diffusion into the ZrO_2_, preventing the formation of a continuous barrier. This was proposed by Seo et al.^16^ and is supported by two key observations. First, the GIXRD measurements show crystal grain sizes around 10 nm, which is larger than the thickness of the individual ZrO_2_ layers. These large crystal grains also cause the rough film surface visible in the TEM image. Second, the Y-rich interlayers revealed in the TEM images are faint compared to the Al_2_O_3_ interlayers. The Y_2_O_3_:ZrO_2_ laminates are crystalline and the lattice planes are very distinct in the TEM image (Figure 3b). The high amount of ZrO_2_ within the material determines the crystalline lattice. In the Y-rich layers, Y atoms occupy the lattice sites of the Zr atoms. This was shown before in.^43,44^ Although the Y_2_O_3_ interlayers are indistinct, they do influence the electrical properties. The E_BD_ values for the 25:5 and 75:5 laminates are higher than for pure ZrO_2_ (Figure 2). For all Y_2_O_3_:ZrO_2_ laminates, the *E*_BD_ is higher than for pure Y_2_O_3_, which was measured as a reference. This phenomenon demonstrates the successful implementation of the thin film properties for the laminated stacks: Although both materials possess a relatively low *E*_BD_ when deposited as a bulk, a lamination of Y_2_O_3_ and ZrO_2_ thin films can exhibit a higher *E*_BD_.

The La_2_O_3_ nanolaminates exhibit only a slightly enhanced *T*_crys_. The TEM image of the 25:5 ZrO_2_:La_2_O_3_ sample shows large crystal grains. At the same time, the GIXRD measurement indicates that these few big grains are embedded in a partially amorphous matrix with many small grains (Figure 3c). This suggests that the nucleation has begun, but the crystal growth has not yet saturated.^42^ No La-rich interlayers are visible in the TEM image, which could be due to two reasons: First, the La deposition rate on ZrO_2_ might be very low, implying that insufficient La_2_O_3_ was deposited to form a dense La_2_O_3_ interlayer. However, this seems unlikely given the clear trend in *E*_BD_, indicating that enough La_2_O_3_ was deposited to alter the electrical behavior. Second, the diffusion of La into the ZrO_2_ layer is even stronger than that for Y, leading to an almost homogeneous distribution of the La atoms within the ZrO_2._ This could explain why they are not visible in TEM imaging. Since La atoms have even larger radii than Y atoms, this diffusion process might be more pronounced.^45^ In this case, this sample could be considered a La-doped ZrO_2_ rather than a true nanolaminate, which also influences *T*_crys_ and *E*_BD_.^40^ The *E*_BD_ increases gradually with the number of La_2_O_3_ interlayers, with an exception for the 40:5 laminate. The 25:5 sample exhibits an *E*_BD_ of 6.6 MV/cm compared to 6.2 MV/cm for pureZrO_2_ (Figure 1).

**Figure 4:**
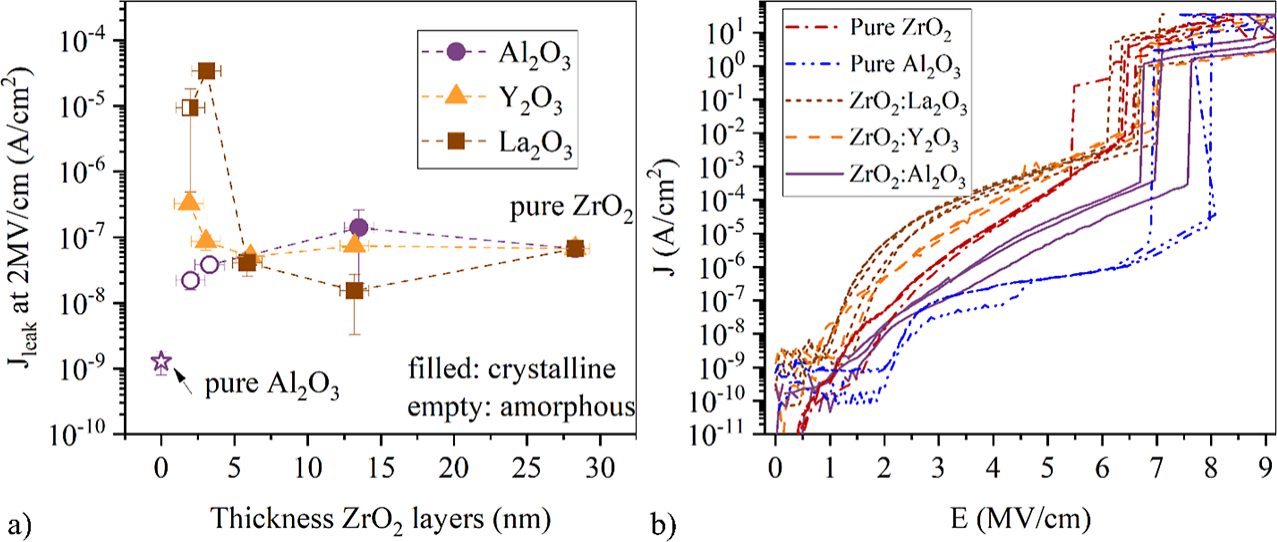
(a) Leakage current density *J*_leak_ evaluated at 2 MV/cm as a function of the thickness of the ZrO_2_ layers within the laminate. A sample with pure Al_2_O_3_ was added for reference. (b) *J*(*E*) curves of all 25:5 nanolaminates and layers formed from pure materials. The strong variation in shape of the *J*(*E*) curves indicates a difference in leakage current mechanisms.

The Al_2_O_3_ nanolaminates exhibit a reduced *J*_leak_ that gradually decreases with increasing Al_2_O_3_ content (Figure 4a). For the 25:5 ZrO_2_:Al_2_O_3_ sample, *J*_leak_ is 2 orders of magnitude lower than for pure ZrO_2_. This reduction can be attributed to the blocking capability of the Al_2_O_3_ interlayers due to the large bandgap of Al_2_O_3_ and the amorphous structure of the stack. In contrast, the bandgap of Y_2_O_3_ and La_2_O_3_ is in the same range as for ZrO_2_ (Table 1), hence these materials cannot increase the electrical barrier for the charge carrier transport. Additionally, the Y_2_O_3_ and La_2_O_3_ laminates are crystalline with Y and La atoms occupying the lattice sites of the Zr atoms. Zr is four-valent but Y and La, in contrast, are trivalent. This induces oxygen vacancies and dangling bonds within the lattice. Such defects promote the leakage through the material, which explains the high *J*_leak_ values for the samples with high Y_2_O_3_ and La_2_O_3_ content.

The leakage mechanism can be understood by examining the current density as a function of the applied electric field (*J*(*E*)) of the 25:5 laminates (Figure 4b). The general shape of the *J*(*E*) curves differs a lot for the different laminates. Unlike the ZrO_2_:Al_2_O_3_ samples, the other laminates show a rapid increase in *J*_leak_ at low voltages, resulting in high *J*_leak_ values at the evaluation field of 2 MV/cm. This suggests that high amounts of Y or La atoms, because of their larger size, disrupt the dense packing of the ZrO_2_ and allow more leakage paths.^16^ This is supported by the TEM images of the 25:5 samples presented in Figure 3, which show a lack of complete interlayers for both laminates. With lower Y or La content, this effect diminishes, and the interlayer formation reduces the leakage compared to pure ZrO_2_. An upper limit for *J*_leak_, generally accepted in literature, is 100 nA/cm^2^ at an applied field of 2 MV/cm.^46^ The ZrO_2_:Al_2_O_3_ laminates and pure Al_2_O_3_ satisfy this criterion.

**Figure 5:**
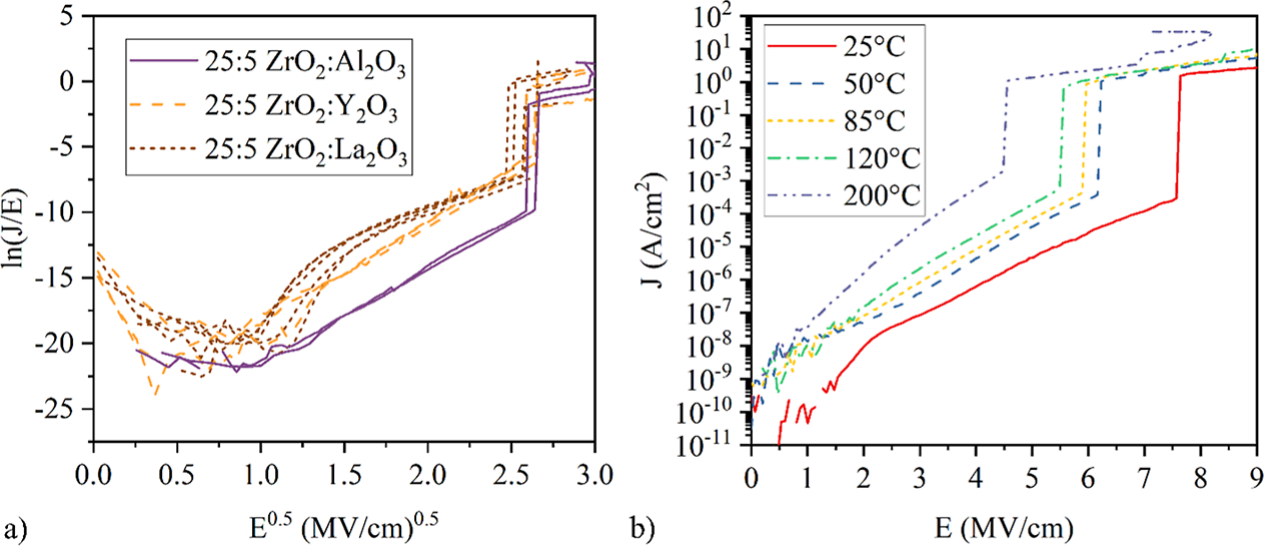
(a) Poole Frenkel plot of the nanolaminates with ZrO_2_:interlayer cycle ratio of 25:5. (b) *J*(*E*) curves of the 25:5 ZrO_2_:Al_2_O_3_ laminate measured at different temperatures between 25 and 200 °C revealing a decline of the electrical breakdown strength and an increase of the leakage at elevated temperatures.

**Figure 6:**
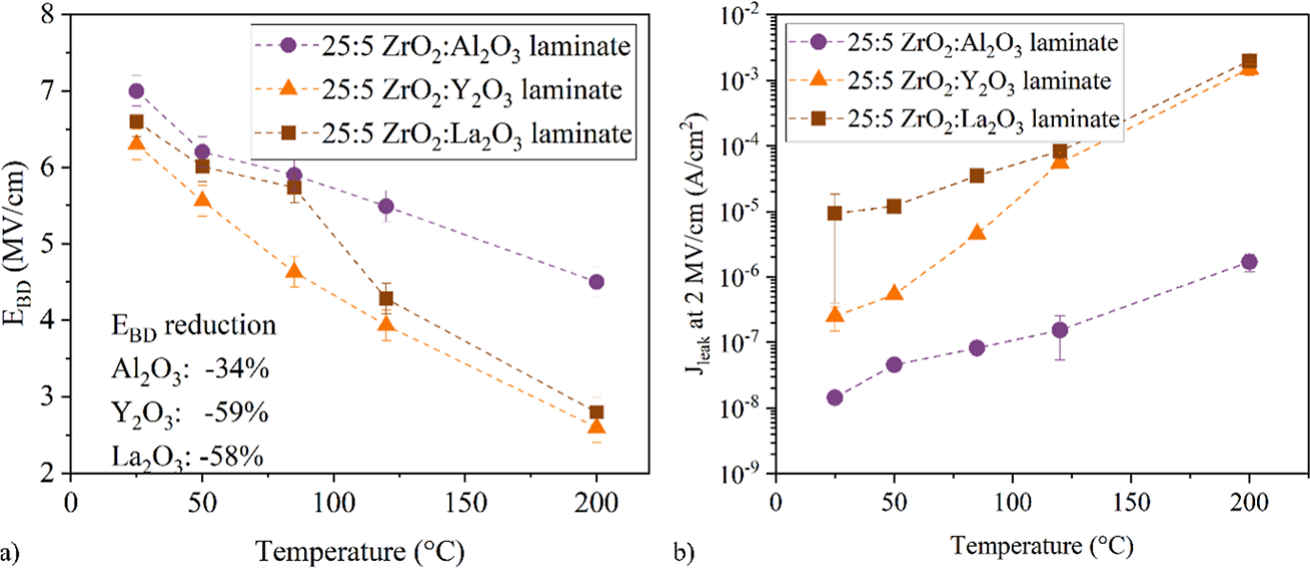
Breakdown field *E*_BD_ (a) and leakage current *J*_leak_ evaluated at 2 MV/cm (b) extracted from temperature dependent *I*(*V*) measurements for the nanolaminates with a cycle ratio of 25:5.

Figure 5a shows the Poole Frenkel plot of the 25:5 nanolaminates, which provides insights into the carrier conduction within the nanolaminated oxide. The curves for ZrO_2_, Al_2_O_3_ and ZrO_2_:Y_2_O_3_ are linear between 1.2 (MV/cm)^0.5^ and 2.5 (MV/cm)^0.5^ for *E*^0.5^, which indicates that the Poole Frenkel mechanism is the dominant leakage mechanism at applied fields between 1.5 and 7 MV/cm. In contrast, the curve for the ZrO_2_:La_2_O_3_ laminates becomes linear at a value of 1.6 (MV/cm)^0.5^ for *E*^0.5^, which corresponds to an applied field of 2.8 MV/cm. This suggests that Poole Frenkel transport becomes dominant at higher fields compared to the other laminates. Southwick et al. proposed that in high-*k* materials multiple leakage mechanisms coexist and superpose each other, with traps and defects playing a significant role.^47^ Our samples exhibit the same behavior, as supported by the temperature-dependent *J*(*E*) characteristics shown in Figure 5b. At elevated temperatures, the leakage current density increases, and the breakdown field decreases (Figure 6). This degradation at higher temperatures is smaller for the Al_2_O_3_ laminates as Al_2_O_3_ is the interlayer material with the highest temperature stability. For these laminates, the *E*_BD_ is reduced by 34% for the measurements at 200 °C compared to the measurements at 25 °C, in contrast to the La_2_O_3_ and Y_2_O_3_ nanolaminates, where the reduction of the breakdown fields is approximately 60%.

### 3.2 Capacitance–Voltage Measurements *C*(*V*)

Capacitance measurements in the accumulation regime reveal the effective *k* value of the dielectric stack including the SiO_2_ film. Assuming the SiO_2_ film and the high-*k* dielectric function as two serial capacitors, the *k* value of the ZrO_2_ based material can be calculated from the *k* value of SiO_2_ and the thicknesses of the SiO_2_ and the high-*k* film. Since La_2_O_3_ exhibits the highest *k* values of all three investigated interlayer materials, closely matching the *k* value of ZrO_2_ (Table 1), it is not surprising the ZrO_2_:La_2_O_3_ laminates exhibit the highest *k* value of all the laminated stacks independent of the amount of La_2_O_3_ within the stack (*k* ∼ 22–23). For the ZrO_2_:Al_2_O_3_ laminates the *k* value decreases gradually with increasing Al_2_O_3_ amount in the stack. This trend is anticipated based on the *k* values of the pure materials presented in Table 1. The 25:5 ZrO_2_:Al_2_O_3_ sample containing approximately 30% Al, has a *k* value of 16.6. This translates to an effective *k* value of 13.6 for the entire stack including the SiO_2_ film (*k*_stack_). The *k* values of the ZrO_2_:Y_2_O_3_ laminates lay in between the *k* values of other two laminate materials with *k* = 18.1 for the 25:5 ZrO_2_:Y_2_O_3_ laminate.

**Figure 7:**
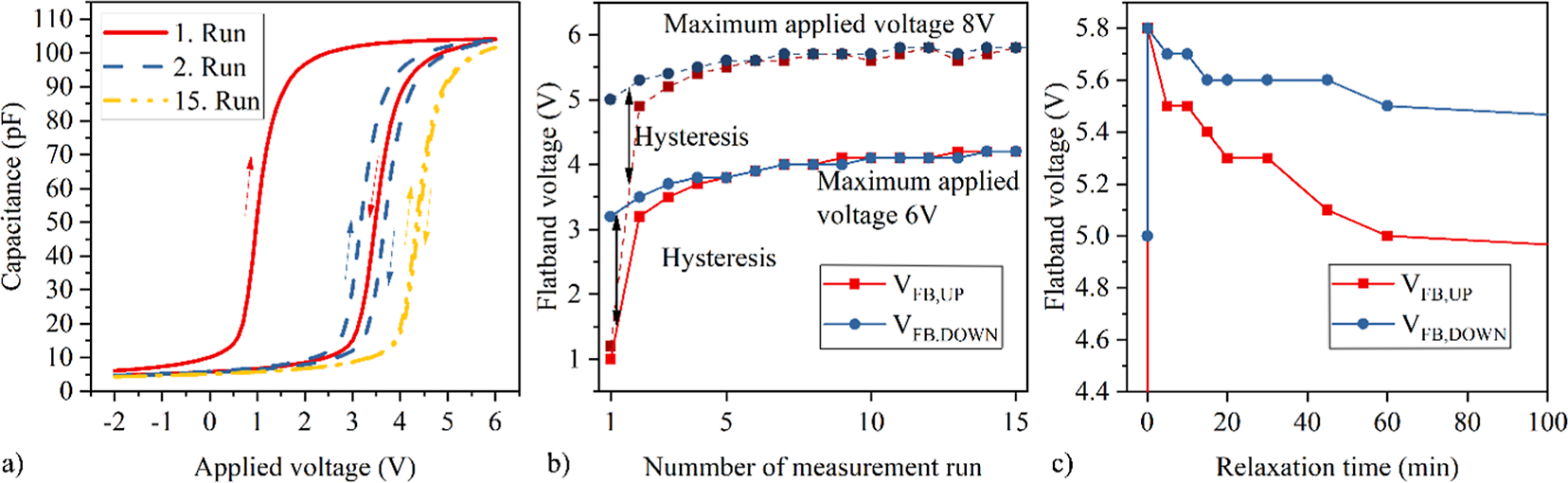
(a) Exemplary *C*(*V*) curves for up- and downsweep, (b) flatband voltage *V*_FB_ as a function of measurement runs and (c) flatband voltage *V*_FB_ as a function of relaxation time for the 25:5 ZrO_2_:Al_2_O_3_ sample.

Nanolamination of binary oxides significantly increases the number of interfaces in the stack, raising concerns about increased charge trapping due to an increased number of interface states. To address this issue, we evaluated the hysteresis of *C*(*V*) measurements. The hysteresis size is determined as the deviation between the flatband voltages in the voltage upsweep *V*_FB,up_ (toward accumulation), and downsweep *V*_FB,down_ (toward depletion). A deviation in the flatband voltage (*V*_FB_) toward positive voltages, i.e. *V*_FB,up_ > *V*_FB,down_, indicates an accumulation of negative charges. That means electrons from the SiC accumulation layer are trapped at the interfaces or in the dielectric layers.^8,18^ Consecutive *C*(*V*) measurements show that the shift in *V*_FB_ remains after the measurement (Figure 7). This suggests that trapped charges do not get released during the downsweep. Furthermore, the shift saturates after a certain number of measurements, which can have two reasons: First, the trapped electrons generate an electric field directed against the initial field which impedes further electron injection. Second, all accessible traps, up to a certain energy level corresponding to the applied bias, are filled. The maximum applied bias defines up to which energy level the traps can be filled, resulting in a larger hysteresis for higher maximum applied voltages. Time-dependent *C*(*V*) measurements reveal that the time constants for detrapping are extremely long (Figure 7c). This trapping behavior poses a potential reliability concern for devices containing such laminates, as the *V*_FB_ shift corresponds to a shift in the threshold voltage *V*_th_. Therefore, reducing the trap density has a high priority.

**Figure 8:**
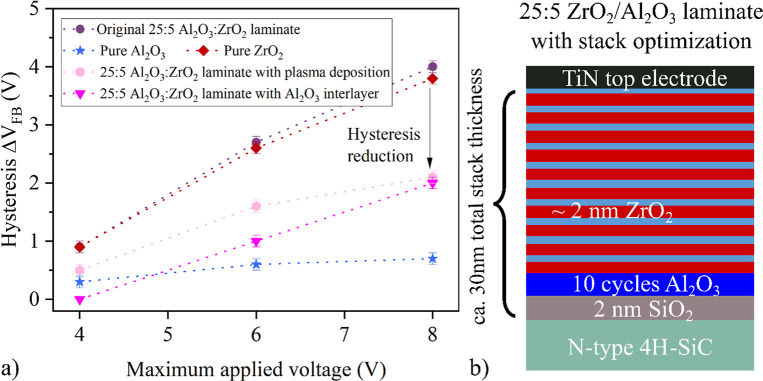
(a) Hysteresis of the nanolaminates depending on the maximum applied bias. By optimizing the deposition conditions and interface of the 25:5 ZrO_2_:Al_2_O_3_ stack its hysteresis could be reduced by 50%. (b) Schematic representation of the optimized stack.

Figure 8 shows that the hysteresis is independent of the number of interlayers in the nanolamination and is similar in size to that of pure ZrO_2_. This suggests that the additional interfaces created due to the lamination do not increase the charge trapping compared to pure ZrO_2_. Furthermore, it indicates that the majority of electrons are trapped at the interface between ZrO_2_ and SiO_2_, especially since the hysteresis of reference samples with pure Al_2_O_3_ is relatively small. For a capacitor with 100 pF and 30,000 μm^2^ electrode size, a hysteresis of 4 V corresponds to a number of 8.3 × 10^12^ trapped electrons per cm^2^. Two methods were explored to implement nanolaminates with smaller hysteresis. First, the entire stack was deposited using O_2_ plasma instead of O_3_ as the oxidant. This approach improved the SiO_2_:ZrO_2_ interface and reduced the hysteresis by 50% compared to the previous stack. However, it also increased *J*_leak_ by 2 orders of magnitude, likely due to the formation of oxygen vacancies, which facilitate charge carrier transport. The high leakage makes this sample unsuitable for further use.

The second approach involved introducing an additional Al_2_O_3_ interlayer between the SiO_2_ and the first very ZrO_2_ layer. This layer was deposited with O_2_ plasma, O_3_ or H_2_O as the oxidant, with the O_2_ plasma-deposited Al_2_O_3_ interlayer showing the best results. Additionally, the last layer of the stack below the TiN top electrode was switched from ZrO_2_ to Al_2_O_3_. A schematic representation of the stack is shown in Figure 8b. For this optimized stack, the hysteresis was reduced by 50% compared to the original stack, which can be attributed to a combination of several aspects: (i) the SiO_2_:ZrO_2_ interface is eliminated and replaced by a SiO_2_:Al_2_O_3_ interface with less traps; (ii) the traps within the ZrO_2_ are physically further away from the semiconductor increasing the barrier thickness for charge trapping; (iii) the band offset between SiC and Al_2_O_3_ is higher than for SiC:ZrO_2_ increasing the barrier height for electrons tunneling from the SiC into the oxide; (iv) the Al_2_O_3_ capping layer of the stack below the top electrode inhibits ZrO_2_ from reacting with TiN to TiO resulting in a barrier lowering for electrons tunneling from the electrode into the oxide.^48^ The resulting stack exhibits a 50% reduction of the hysteresis, an *E*_BD_ almost as high as for pure Al_2_O_3_ (7.4 MV/cm), an acceptably low *J*_leak_ of 1.0 × 10^–7^ A/cm^2^, and a *k* value of 13. The beneficial effect of the Al_2_O_3_ film was also observed by Besling et al. in Si substrates.^14^ Surprisingly, the measured *J*_leak_ was slightly higher than that of the laminates without this Al_2_O_3_ interlayer, which is not yet fully understood, but *J*_leak_ remains within the acceptable limit of 1 × 10^–7^ A/cm^2^.^46^ In summary, this stack demonstrates the most suitable performance.

## 4 Discussion

Among the three interlayer materials investigated, Al_2_O_3_ enables the most suitable performance. The best presented stack is the 25:5 ZrO_2_:Al_2_O_3_ laminate featuring an O_2_ plasma-deposited Al_2_O_3_ interlayer between the SiO_2_:ZrO_2_ interface, and capped with a Al_2_O_3_ film. This stack demonstrates a high electrical breakdown field of 7.4 MV/cm, a dielectric constant of 13 and *J*_leak_ of 1.0 × 10^–7^ A/cm^2^, which is within the acceptable limit. The introduction of the O_2_ plasma-deposited Al_2_O_3_ interfacial layer and the Al_2_O_3_ capping reduces the hysteresis by 50% compared to the original stack. Alternatively stated, the presented stack demonstrates electrical parameters that are close to that of Al_2_O_3_ while maintaining a *k* value that is twice as high. The stack with the lowest leakage current is the Al_2_O_3_ nanolaminate without an Al_2_O_3_ interfacial layer with *J*_leak_ 2.2 × 10^–8^ A/cm^2^. The enhanced electrical breakdown field and the reduced leakage of the ZrO_2_:Al_2_O_3_ laminates can be attributed to the amorphous structure of these sample. The Al_2_O_3_ interlayers completely interrupt the ZrO_2_ bulk and thereby increase the crystallization temperature. The 25:5 ZrO_2_:Al_2_O laminate enables the highest *T*_crys_ of 750 °C. Y_2_O_3_ and La_2_O_3_ interlayers did not affect *T*_crys_ and only slightly improved the electrical parameters of the ZrO_2_-based stacks.

**Figure 9:**
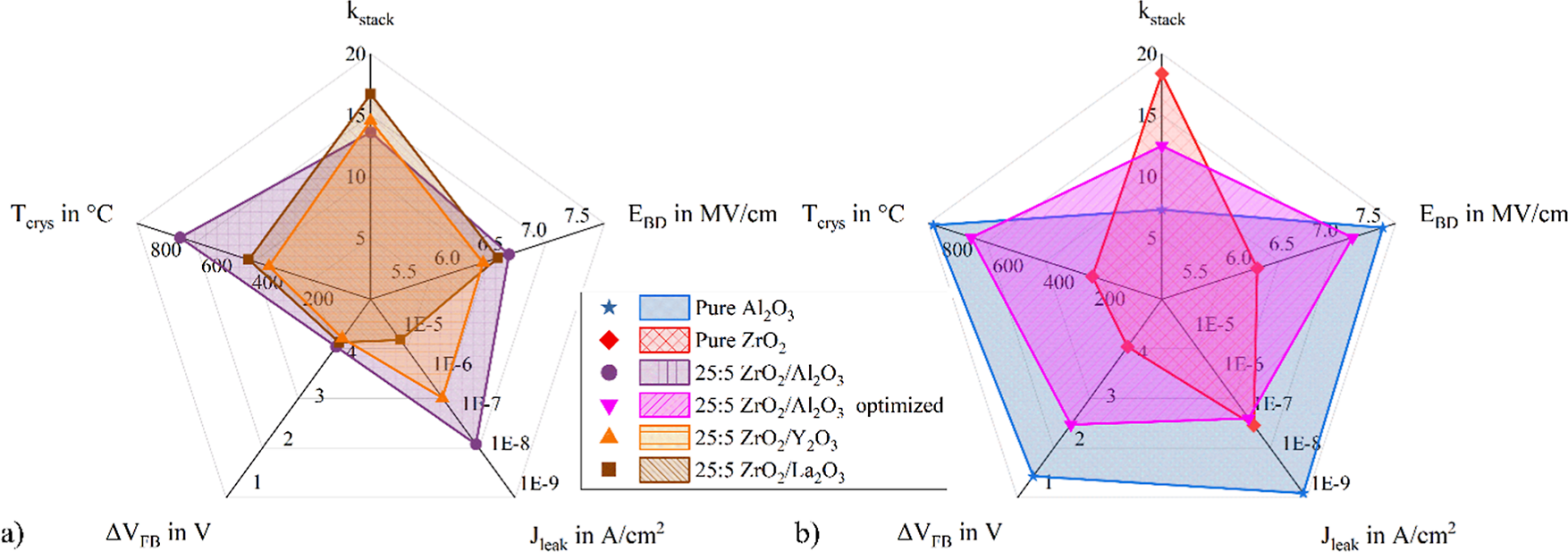
Spider graphs comparing the performance parameters of all 25:5 laminates. (a) Spider graphs for the 25:5 ZrO_2_:Al_2_O_3_, 25:5 ZrO_2_:Y_2_O_3_ and 25:5 ZrO_2_:La_2_O_3_ laminates with Al_2_O_3_ interlayers outperforming Y_2_O_3_ and La_2_O_3_ interlayers. (b) Spider graphs for pure ZrO_2_, pure Al_2_O_3_ and the optimized ZrO_2_:Al_2_O_3_ laminate, which is a suitable compromise between ZrO_2_ and Al_2_O_3_.

A comparison of all the mentioned key parameters for the 25:5 laminates is shown in Figure 9. The axes of the graphs are defined such that the least favorable values are centralized. The desired metrics, i.e. high *E*_BD_ and low *J*_leak_, are positioned furthest from the center. Hence, a larger area encompassed by a graph corresponds to more favorable parameters. All nanolaminates strike a balance between electrical strength and high dielectric constant. The Al_2_O_3_ laminates exhibit superior overall performance, which is further enhanced by the mentioned stack adjustments. The optimized stack effectively reconciles the benefits of pure ZrO_2_ and pure Al_2_O_3_, offering an appropriate compromise.

**Figure 10:**
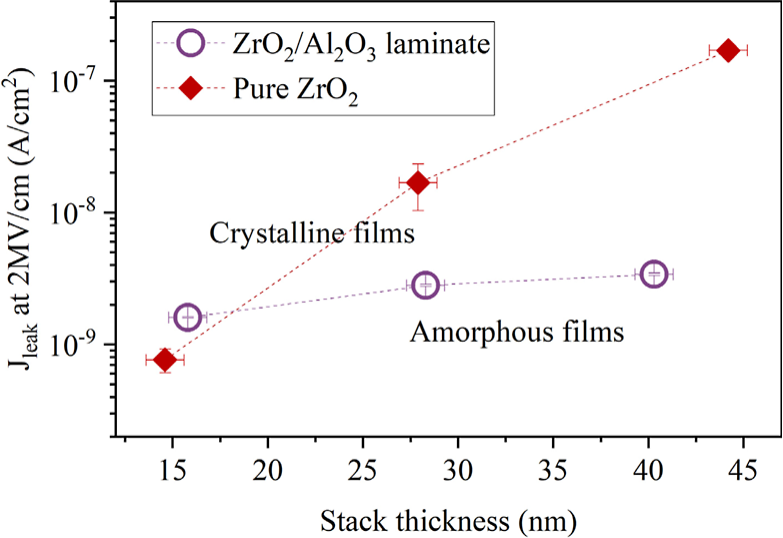
Leakage current density at 2 MV/cm as a function of stack thickness on Si substrates. In contrast to the leakage for pure ZrO_2_, *J*_leak_ for the ZrO_2_:Al_2_O_3_ nanolaminates remains within the same order of magnitude when increasing the stack thickness.

For a final assessment of the leakage current reduction in thick ZrO_2_-based stacks, the most promising stack, namely the 25:5 ZrO_2_:Al_2_O_3_ laminate, was investigated as a function of stack thickness. Therefore, the 25:5. ZrO_2_:Al_2_O_3_ laminate and pure ZrO_2_ stack were deposited with a thickness of up to 43 nm on silicon reference samples with a 6 nm thick SiO_2_ layer. In contrast to the samples with pure ZrO_2_, the 25:5 ZrO_2_:Al_2_O_3_ laminate exhibits only a slight increase in *J*_leak_ when scaling up the stack thickness. This can be associated with the amorphous structure of the laminates, contrary to the crystalline ZrO_2_. Although *J*_leak_ for the laminates is slightly higher than that for a 15 nm ZrO_2_ film, it remains below the acceptable limit. The key achievement is that *J*_leak_ for the 43 nm thick film lies within the same range as for the 15 nm film (Figure 10). This demonstrates that Al_2_O_3_ interlayers can maintain the leakage current at an acceptably low level when the thickness of the ZrO_2_-based films is increased.

Compared to ZrO_2_ based stacks in the current literature, our ZrO_2_:Al_2_O_3_ laminates exhibit an increase in dielectric strength and crystallization temperature while maintaining a high dielectric constant. Similar values have been reported by Lo Nigro et al., who fabricated HfO_2_:Al_2_O_3_ laminates with a k value of 12.4 and a *T*_crys_ > 800 °C.^13^ ZrO_2_:Al_2_O_3_ laminates on Si substrates have been presented by Besling et al. with k values similar to our materials. In agreement with our results, they state that charge trapping could be reduced by depositing Al_2_O_3_ as a first layer on the substrate instead of ZrO_2_.^14^ However, both reports do not provide information about the electric breakdown field of their materials.

## 5 Conclusion

In this study, we successfully demonstrated that the incorporation of Y_2_O_3_, La_2_O_3_ and Al_2_O_3_ interlayers can significantly enhance the electric breakdown field, reduce the leakage current and increase the crystallization temperature of ZrO_2_-based dielectrics. The most promising results were achieved with Al_2_O_3_ interlayers. The crystallization temperature was increased from 375 °C for pure ZrO_2_ up to 750 °C for the 25:5 ZrO_2_:Al_2_O_3_ laminate. In contrast to pure ZrO_2_, the leakage for ZrO_2_:Al_2_O_3_ laminates remains within the acceptable limit when increasing the stack thickness up to 45 nm. Thus, we presented an effective approach to mitigate high leakage in thick ZrO_2_-based dielectrics. Furthermore, by introducing an additional plasma-deposited Al_2_O_3_ interlayer between the SiO_2_ and ZrO_2_ films and concluding the stack with Al_2_O_3_ instead of ZrO_2_, we achieved a reduction of the charge trapping by 50% compared to the original stack. The optimized ZrO_2_:Al_2_O_3_ stack exhibits an *E*_BD_ of 7.4 MV/cm and a k value of 13, thereby demonstrating an electrical strength comparable to Al_2_O_3_ while maintaining a *k* value twice that of Al_2_O_3_.
